# MicroRNA-449a maintains self-renewal in liver cancer stem-like cells by targeting *Tcf3*

**DOI:** 10.18632/oncotarget.22705

**Published:** 2017-11-27

**Authors:** Qianzhen Zhang, Zhi Yang, Juanjuan Shan, Limei Liu, Chungang Liu, Junjie Shen, Xuejiao Chen, Yanmin Xu, Jun Chen, Qinghua Ma, Li Yang, Cheng Qian

**Affiliations:** ^1^ Key Laboratory of Biorheological Science and Technology, Ministry of Education, Bioengineering College, Chongqing University, Chongqing, 400044, China; ^2^ College of Bioengineering, Chongqing University, Chongqing, 400044, China; ^3^ Center of Biological Therapy, Southwest Hospital, Third Military Medical University, Chongqing, 400038, China

**Keywords:** cancer stem cells, MicroRNA-449a, self-renewal, Nanog, TCF3

## Abstract

Cancer stem cells (CSCs) are thought to be responsible for tumor invasion, metastasis, and recurrence. We previously showed that the pluripotency factor Nanog not only serves as a novel biomarker of CSCs but also potentially plays a crucial role in maintaining the self-renewal ability of liver CSCs. However, how CSCs maintain *Nanog* gene expression has not been elucidated. Here, we demonstrated that microRNA-449a (miR-449a) is overexpressed in poorly differentiated hepatocellular carcinoma tissues, drug-resistant liver cancer cells, cultured liver tumorspheres, and Nanog-positive liver cancer cells. The upregulation of miR-449a in non-CSCs increased stemness, whereas the downregulation of miR-449a in Nanog-positive CSCs reduced stemness. Furthermore, transcription factor 3 (TCF3), a target of miR-449a, could downregulate *Nanog* expression, and restoring TCF3 expression in miR-449a-expressing Nanog-negative cells abrogated cellular stemness. These data establish that the miR449a-TCF3-Nanog axis maintains stemness in liver CSCs.

## INTRODUCTION

Hepatocellular carcinoma (HCC), one of the most lethal cancers, can be effectively treated with surgical resection and liver transplantation [1]; however, recurrence and metastasis after these procedures remain primary obstacles for liver cancer treatment [2–4]. Cancer stem cells (CSCs), or tumor-initiating cells, have been identified in multiple malignancies, including liver cancer [5, 6]. These cells have unique characteristics and are thought drive tumor initiation, development, metastasis and recurrence [6–8]. As such, CSCs represent novel therapeutic targets for the treatment and prevention of tumor progression. The identification of CSC-specific markers, the isolation and characterization of CSCs from malignant tissues, and targeting strategies for the destruction of CSCs have provided new opportunities for cancer research [8, 9]. We previously demonstrated that the pluripotency factor Nanog not only serves as a novel biomarker for CSCs but also potentially plays a crucial role in maintaining the self-renewal capability of liver CSCs [10]. However, despite the potential clinical importance, the regulation of Nanog in CSCs at the molecular level is not well understood.

MicroRNAs (miRNAs), a class of endogenous non-coding RNAs that help regulate several cellular processes [11, 12], appear to have a role in tumor initiation through their regulation of CSC properties such as self-renewal, tumorigenicity and drug resistance [13–16]. The miR-449 cluster, which belongs to the miR-34 family and encodes the highly conserved miRNAs including miR-449a, miR-449b and miR-449c [17], has been shown to cause cell cycle arrest or promote apoptosis in a variety of cell and cancer types [18–20]. In addition, miR-449 expression is decreased in many tumor tissues [19, 21, 22]. However, to date, no studies have directly demonstrated that miR-449 has a causal role in tumorigenesis, despite the decrease in miR-449 levels in tumor tissues. Furthermore, the biological functions of miR-449 in CSCs are unknown. Therefore, obtaining a better understanding of how miR-449 regulates the expression of genes related to stemness could provide new insights into the process of tumorigenesis and reveal new therapeutic targets.

Here, we demonstrated that miR-449a is upregulated in HCC tissues and that high expression of miR-449a correlates with poor clinical prognosis. Furthermore, we showed that miR-449a participates in CSC regulation by improving self-renewal ability. Exogenous expression of miR-449a increased the stem cell properties of HCC cell lines and enhanced drug resistance. Moreover, increased expression of miR-449a resulted in higher expression of *Nanog* and downregulation of the *Tcf3* gene, which negatively regulates Nanog, and TCF3 was confirmed as a novel target of miR-449a. These findings indicate that miR-449a has important roles in the self-renewal and tumor progression of CSCs. Collectively, this study reveals the miR-449a–TCF3-Nanog axis as a potential therapeutic target for liver cancer.

## RESULTS

### Upregulation of miR-449 in human HCC is correlated with poor prognosis

**Figure 1:**
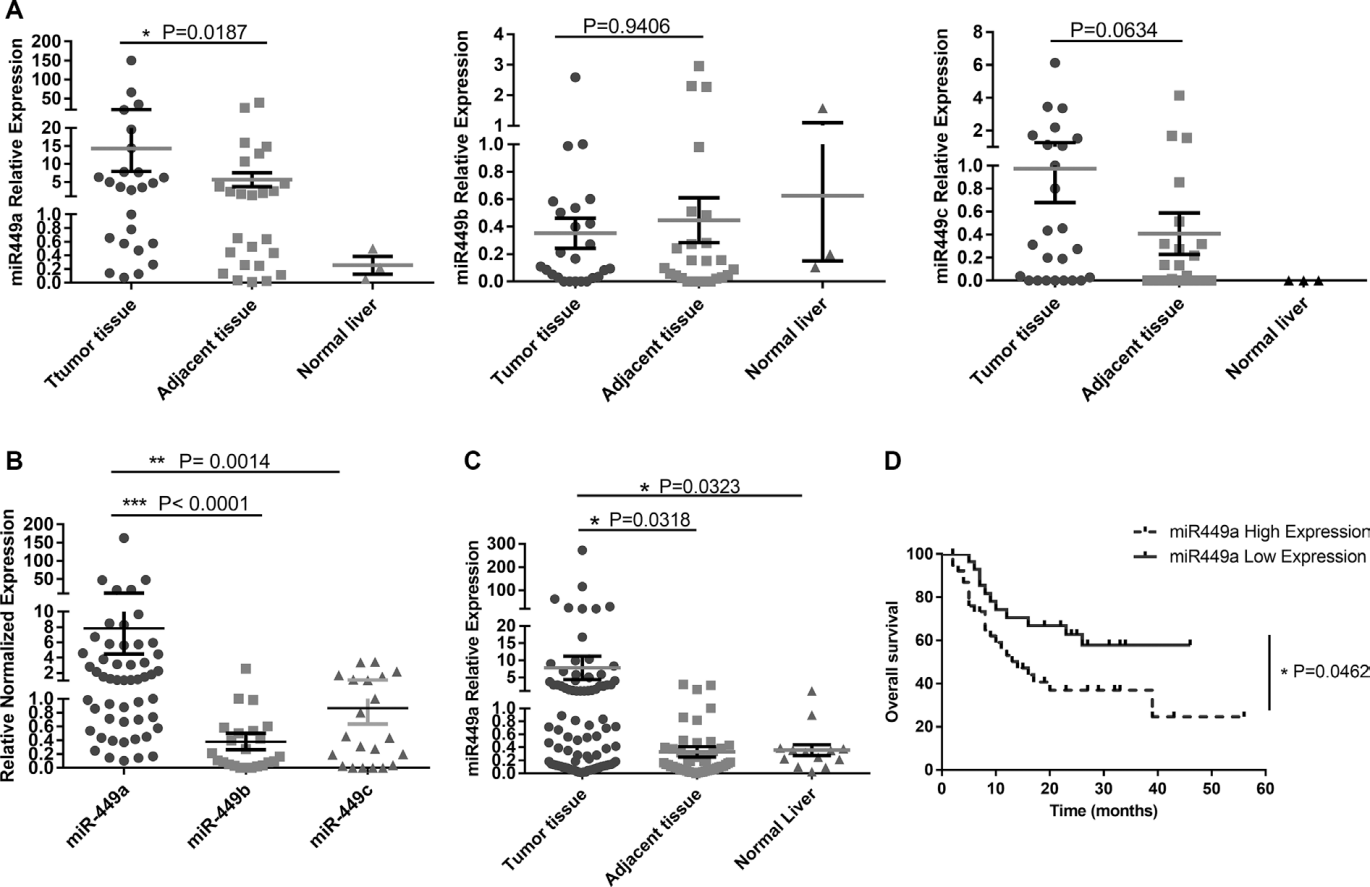
miR-449 is upregulated in HCC patients and correlated with poor prognosis (**A**) Relative expression of miR-449 (a/b/c) was measured in 25 fresh HCC tumor tissues, paired adjacent tissues and 3 normal liver tissues using qRT-PCR. (**B**) Comparison of miR-449a, miR-449b and miR-449c expression in HCC tissue; miR-449a expression was much higher than that of the other miR-449 subtypes. (**C**) Relative expression of miR-449a in a large set of patient samples, which included fresh HCC tissue samples paired with adjacent normal tissues (*n* = 52), paraffin-embedded tissue sections (*n* = 36), and normal liver tissues (*n* = 13). (**D**) Expression of miR-449a correlated with poor overall survival of human HCC patients. HCC patients were divided into two groups: a low-miR-449a-expression group (miR-449a expression levels below the median, *n* = 37, solid line) and a high-miR-449a-expression group (miR-449a expression levels above the median, *n* = 38, dotted line). Overall survival of these patients is shown. *P* values were generated using the log-rank test.

The miR-449 cluster contains three miRNAs: miR-449a, b, and c. To investigate the expression of the miR-449 cluster in malignant liver cancer, the levels of these miRNAs were measured in 25 fresh HCC tissue samples, paired adjacent normal tissues and three normal liver tissues. The results showed marked upregulation of miR-449a in HCC tissues but no significant differences in the expression of miR-449b and miR-449c (Figure 1A). Furthermore, miR-449a was expressed at much higher levels than miR-449b or miR-449c (Figure 1B). Therefore, we utilized qRT-PCR to analyze the expression of only miR-449a in a larger set of patient samples, including fresh HCC tissue samples paired with adjacent normal tissues (*n* = 52), paraffin-embedded tissue sections (*n* = 36), and normal liver tissues (*n* = 13). The results revealed that miR-449a is upregulated in HCC tissue (Figure 1C). We next analyzed correlations between miR-449a expression and overall survival, tumor recurrence, and other clinical data for 75 patients with follow-up data. Kaplan-Meier analysis and the log-rank test were used to compare the overall survival of HCC patients on the basis of miR-449a expression levels. To accomplish this, the HCC patients were divided into two groups: a low-miR-449a-expressing group (miR-449a expression levels below the median, *n* = 37) and a high-miR-449a-expressing group (miR-449a expression levels above the median, *n* = 38). Remarkably, high expression of miR-449a was directly correlated with poor overall survival (Figure 1D). Subsequently, we examined correlations between miR-449a expression and other clinical parameters. Pathology analysis showed that high levels of miR-449a were significantly associated with tumor recurrence (*P* = 0.013), metastasis (*P* < 0.001), vascular invasion (*P* = 0.009) and interstitial hyperplasia in tumors (*P* = 0.029) (Table 1). The total number was < 75 for several clinical parameters due to missing data.

**Table 1:** Correlation between miR-449a levels in HCC tissues and clinicopathological parameters of HCC patients

		miR-449a		
**Variable**	n	Low	High	*p*-value
**Age (years)**				
< 50	47	22	25	0.577
≥ 50	28	15	13	
**Sex**				
Female	7	3	4	0.723
Male	68	34	34	
**Tumor recurrence**				
−	14	11	3	0.013^*^
+	45	16	29	
**Metastasis**				
−	20	17	3	< 0.001^***^
+	39	10	29	
**Tumor grade**				
**I**	9	2	7	0.068
**II**	34	16	18	
**III**	31	18	13	
**Tumor size (cm)**				
< 5	16	10	6	0.192
≥ 5	57	25	32	
**Tumor features**				
**Necrosis**				
+	51	25	26	0.474
++	17	7	10	
+++	6	4	2	
**Vascular invasion**				
−	12	10	2	0.009^**^
+	55	24	31	
++	7	2	5	
**Interstitial hyperplasia**				
+	54	22	32	0.029^*^
++	14	10	4	
+++	6	4	2	
**Capsular invasion**				
−	19	9	10	0.401
+	35	18	17	
++	20	9	11	

Note: (1) ^*^P < 0.05, ^**^P < 0.01, and ^***^P < 0.001 significant difference. (2) χ^2^ test. (3) Total number < 75 due to missing data.

### miR-449a promotes self-renewal and tumorigenesis in human liver cancer cells

**Figure 2:**
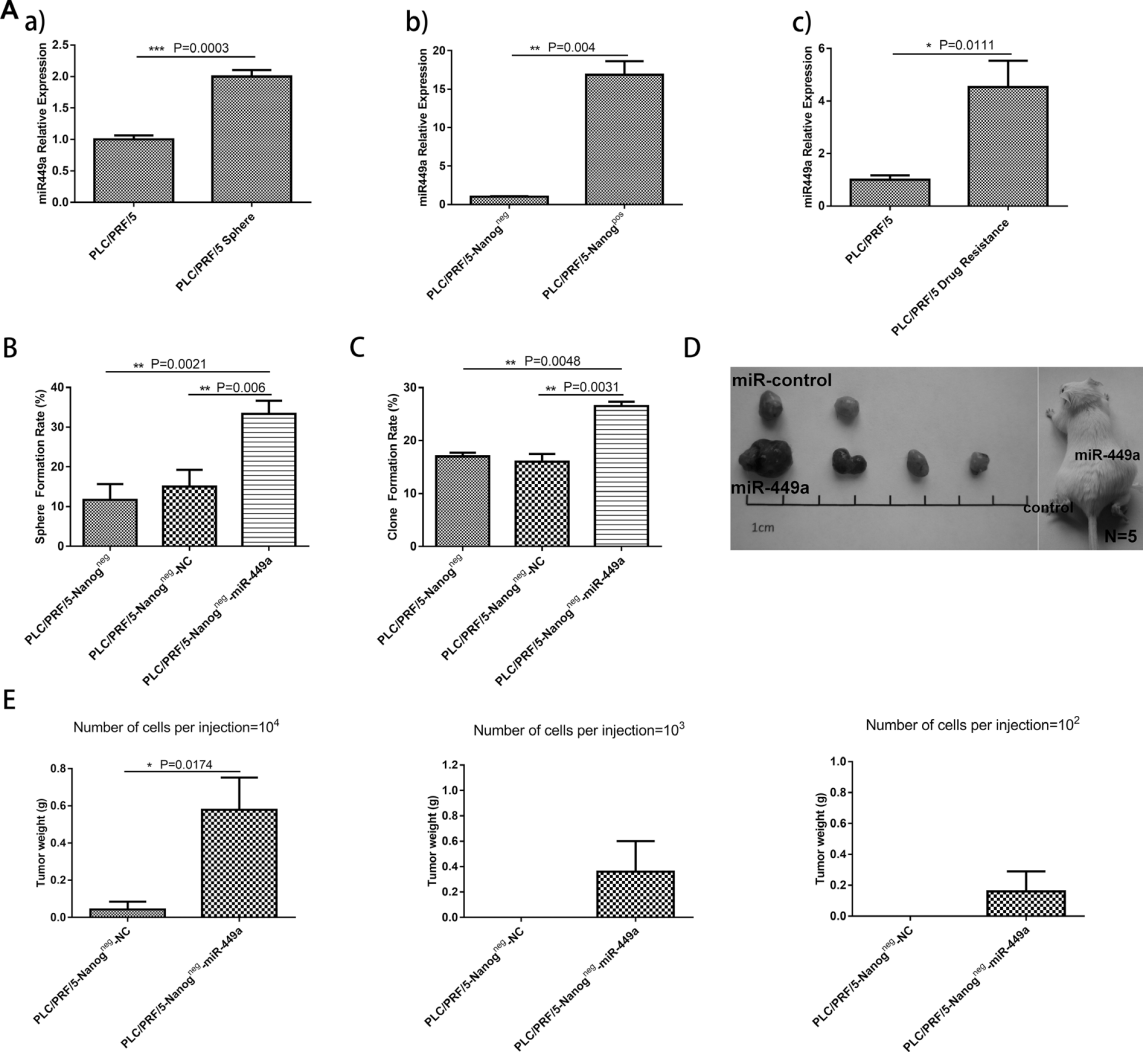
Overexpression of miR-449a promotes self-renewal and tumorigenesis in human liver cancer cells *in vitro* and *in vivo* (**A**) Relative expression of miR-449a in normal cultured cells or cancer stem-like cells was quantified using qRT-PCR. a), Relative expression of miR-449a in PLC/PRF/5 and PLC/PRF/5 sphere cells. miR-449a expression was remarkably increased in the PLC/PRF/5 sphere cells. b**)**, Relative expression of miR-449a in PLC/PRF/5 Nanog^neg^ and Nanog^pos^ cells. miR-449a expression was dramatically increased in the PLC/PRF/5 Nanog^pos^ cells. c), Relative expression of miR-449a in PLC/PRF/5 and PLC/PRF/5 drug-resistant cells. miR-449a expression was significantly increased in the PLC/PRF/5 drug-resistant cells. (The data are presented as the mean ± SD of three independent experiments; ^***^P < 0.001; ^**^P < 0.01; ^*^P < 0.05). (**B**) Sphere formation rate of PLC/PRF/5 Nanog^neg^ cells, scrambled control-expressing PLC/PRF/5 Nanog^neg^ cells (Nanog^neg^-NC) and miR-449a-expressing PLC/PRF/5 Nanog^neg^ cells (Nanog^neg^-miR-449a) grown in suspension culture conditions for 14 days. The sphere formation rate was significantly increased in the Nanog^neg^-miR-449a group. (The data are presented as the mean ± SD of three independent experiments; ^**^P < 0.01). (**C**) Clone formation rate of PLC/PRF/5 Nanog^neg^ cells, scrambled control-expressing PLC/PRF/5 Nanog^neg^ cells (Nanog^neg^-NC) and miR-449a-expressing PLC/PRF/5 Nanog^neg^ cells (Nanog^neg^-miR-449a) grown in conventional culture conditions for 14 days. The clone formation rate was significantly increased in the Nanog^neg^-miR-449a group. (The data are presented as the mean ± SD of three independent experiments; ^**^P < 0.01). (**D**) Effects of miR-449a on tumor formation *in vivo*. A total of 1 × 10^4^ control or miR-449a-overexpressing PLC/PRF/5 Nanog^neg^ cells were injected into NOD-SCID mice (5 mice per group). Representative xenografts from each of the five mice are shown. Lentivirus-mediated overexpression of miR-449a significantly increased tumor formation. (**E**) Average weight of tumors formed from control or miR-449a-overexpressing PLC/PRF/5 Nanog^neg^ cells initiated by implantation of 1 × 10^2^, 1 × 10^3^, or 1 × 10^4^ cells. Overexpression of miR-449a significantly increased tumor weight. (5 mice per group, and the data are presented as the mean ± SD; ^*^P < 0.05).

Previous reports have shown that tumorspheres, drug-resistant cells and Nanog^pos^ cells have more stem cell potential than their counterparts. Therefore, we next measured miR-449a expression in tumorspheres, drug-resistant cells, Nanog^pos^ cells and their counterparts. The results indicated that miR-449a is upregulated in stem-like cells (Figure 2A). To further characterize how miR-449a affects the stemness of liver cancer cells, the following lentiviral vectors were constructed: 1) Lv-miR-449a, which induced the expression of hsa-miR-449a, and Lv-NC, the corresponding scrambled control; and 2) Lv-sh-miR-449a, which induced the short hairpin RNA (shRNA) targeting the 3’UTR of miR-449a, and Lv-sh-NC, the corresponding scrambled control.

**Figure 3:**
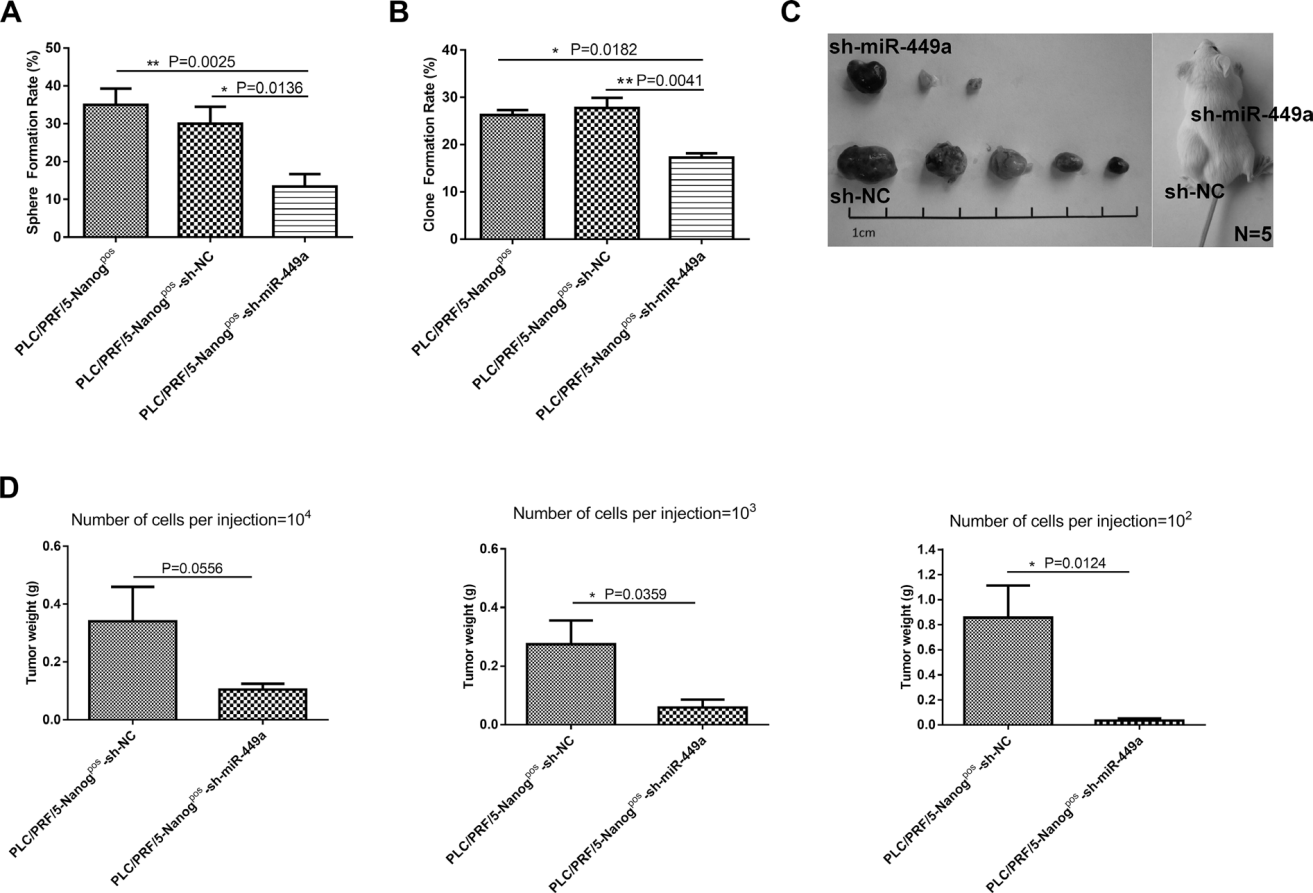
Downregulation of miR-449a inhibited self-renewal and tumorigenesis in human liver cancer cells *in vitro* and *in vivo* (**A**) Sphere formation rate of PLC/PRF/5 Nanog^pos^ cells, scrambled control shRNA-expressing PLC/PRF/5 Nanog^pos^ cells (Nanog^pos^-sh-NC) and miR-449a shRNA-expressing PLC/PRF/5 Nanog^pos^ cells (Nanog^pos^-sh-miR-449a) grown in suspension culture conditions for 14 days. The sphere formation rate significantly decreased in the Nanog^pos^-sh-miR-449a group. (The data are presented as the mean ± SD of three independent experiments; ^**^P < 0.01; ^*^P < 0.05). (**B**) Clone formation rate of PLC/PRF/5 Nanog^pos^ cells, scrambled control shRNA-expressing PLC/PRF/5 Nanog^pos^ cells (Nanog^pos^-sh-NC) and miR-449a shRNA-expressing PLC/PRF/5 Nanog^pos^ cells (Nanog^pos^-sh-miR-449a) grown in conventional culture conditions for 14 days. The clone formation rate significantly decreased in the Nanog^pos^-sh-miR-449a group. (The data are presented as the mean ± SD of three independent experiments; ^**^P < 0.01; ^*^P < 0.05). (**C**) Effects of miR-449a on tumor formation *in vivo*. A total of 1 × 10^2^ control shRNA-expressing or miR-449a shRNA-expressing PLC/PRF/5 Nanog^pos^ cells were injected into NOD-SCID mice (5 mice per group). Representative xenografts from each of the five mice are shown. Lentivirus-mediated downregulation of miR-449a significantly decreased the tumor formation of PLC/PRF/5 Nanog^pos^ cells. (**D**) Average weight of tumors formed from control shRNA-expressing or miR-449a shRNA-expressing PLC/PRF/5 Nanog^pos^ cells initiated by implantation of 1 × 10^2^, 1 × 10^3^, or 1 × 10^4^ cells. Downregulation of miR-449a significantly reduced tumor weight. (5 mice per group, and the data are presented as the mean ± SD; ^**^P < 0.01; ^*^P < 0.05).

After verifying that Nanog^neg^ liver cancer cells have low stemness potential, we investigated whether miR-449a can enhance the self-renewal and tumorigenesis of these cells. To accomplish this, we infected Nanog^neg^ cancer cells with Lv-miR-449a and Lv-NC. The cells overexpressing miR-449a exhibited markedly increased stemness, as indicated by increased sphere formation and colony formation ability and enhanced *in vivo* tumorigenic ability, compared with the Lv-NC-infected cells (Figure 2B–2E, Table 2, Supplementary Figure 1A). In complement to the above, Nanog^pos^ liver cancer cells have been reported to possess high stemness potential. We verified these effects by infecting Nanog^pos^ CSCs with Lv-sh-miR-449a or Lv-sh-NC virus to knock down miR-449a in the cells. The results showed that miR-449a shRNA remarkably inhibited the self-renewal ability of liver CSCs (Figure 3A–3D, Table 2, Supplementary Figure 1B), highlighting the important role of this miRNA in maintaining stemness.

**Table 2:** Regulation of tumorigenesis in subcutaneous xenografts by miR-449a

Cells	Number of cells per injection		
	10^4^	10^3^	10^2^
PLC/PRF/5 Nanog^neg^-NC	2/5	0/5	0/5
PLC/PRF/5 Nanog^neg^-449a	4/5	3/5	2/5
PLC/PRF/5 Nanog^pos^-sh-NC	5/5	5/5	5/5
PLC/PRF/5 Nanog^pos^-sh-miR-449a	5/5	4/5	3/5

Note: Efficiency of tumor formation of scrambled control-expressing PLC/PRF/5 Nanog^neg^ cells (PLC/PRF/5 Nanog^neg^-NC), miR-449a-expressing PLC/PRF/5 Nanog^neg^ cells (PLC/PRF/5 Nanog^neg^-449a), scrambled control shRNA-expressing PLC/PRF/5 Nanog^pos^ cells (PLC/PRF/5 Nanog^pos^-sh-NC) and miR-449a shRNA-expressing PLC/PRF/5 Nanog^pos^ cells (PLC/PRF/5 Nanog^pos^-sh-miR-449a). Varying numbers of cells were subcutaneously injected into NOD-SCID mice (5 mice per group). Tumor formation was observed for 8 weeks after implantation.

### TCF3 is a direct target of miR-449a

**Figure 4:**
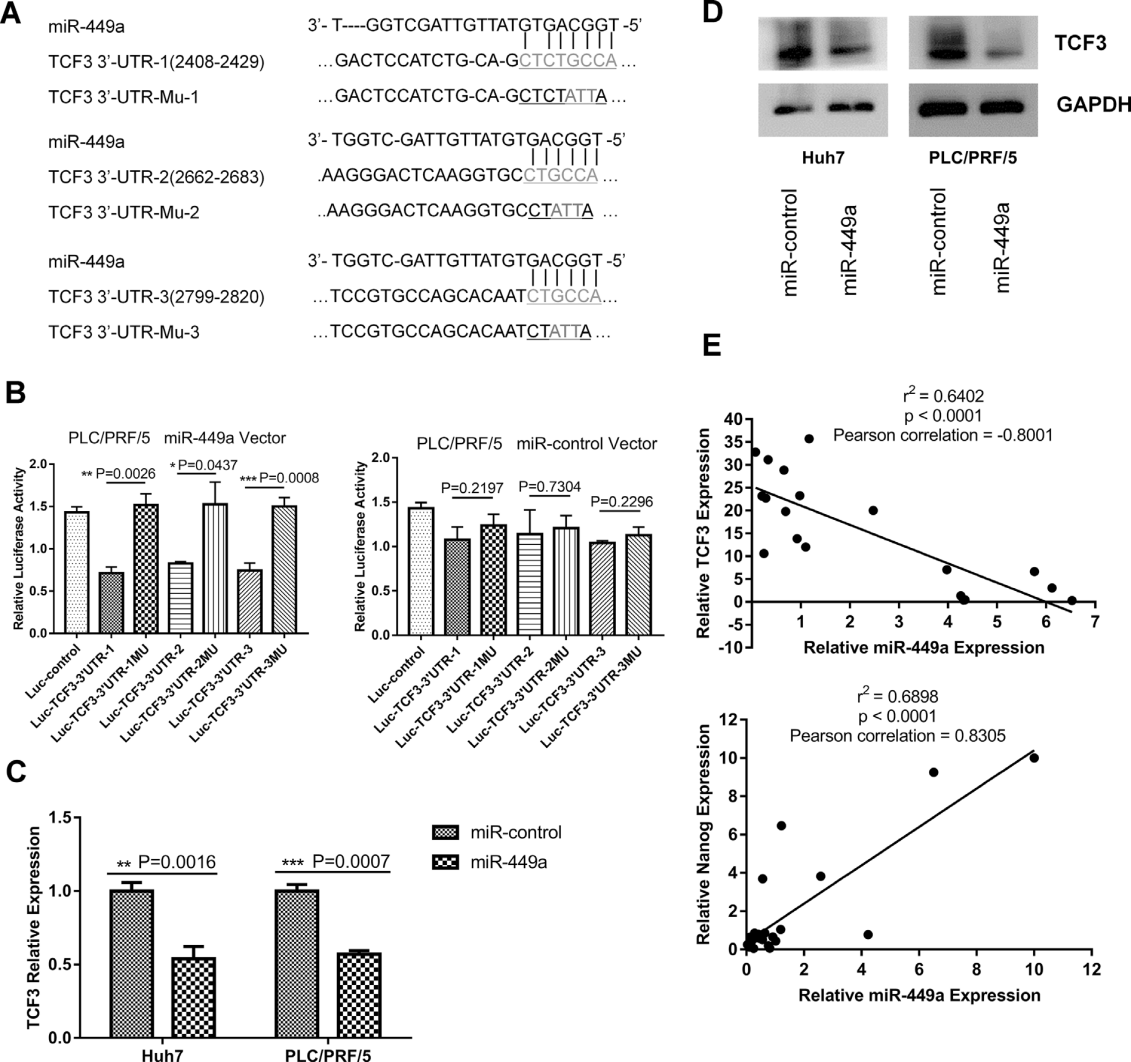
miR-449a targeted Tcf3 by binding to its 3’UTR (**A**) Three predicted miR-449a target sequences in the 3’UTR of *Tcf3* and a mutant sequence (TCF3-3’UTR-Mu) were cloned into pGL3-control vectors. (**B**) Luciferase reporter activity of the *Tcf3* 3’UTR was measured in PLC/PRF/5 cells. PLC/PRF/5 cells were cotransfected with miR-449a or scrambled control and luciferase reporters containing either the predicted miRNA target site in the TCF3 3’UTR or its corresponding mutant form, TCF3-3’UTR-Mu, which was used as a positive control. (The data are presented as the mean ± SD; ^***^P < 0.001; ^**^P < 0.01; ^*^P < 0.05). (**C**, **D**) TCF3 mRNA and protein levels were reduced compared with that in control cells after the infection of Huh7 and PLC/PRF/5 cells with miR-449a-expressing viruses. (**E**) Correlation between miR-449a and TCF3 (upper panel) or Nanog (lower panel) mRNA levels in 25 fresh HCC tissue samples and paired adjacent normal tissues.

To investigate the molecular mechanism driving miR-449a-mediated promotion of stemness in liver cancer cells, bioinformatic algorithms were used to identify potential targets of miR-449a in humans. TCF3 (also known as TCF7L1) was predicted as a target of miR-449a according to RNA22 [23], with three conserved binding sites for miR-449a being identified in the 3’UTR region of the *Tcf3* gene (Figure 4A). To verify the prediction, we constructed three luciferase reporter plasmids containing the predicted miR-449a-binding sites in the 3’UTR of *Tcf3* and three luciferase reporter plasmids containing mutated versions of the binding sites. To further examine whether miR-449a affects TCF3 expression, we infected Huh7 and PLC/PRF/5 cells with Lv-miR-449a and Lv-NC. The targeting activity of miR-449a to the 3’UTRs of the TCF3 mRNA constructs containing the predicted binding sites was examined using luciferase constructs cloned into a pGL3-control vector. For wild-type TCF3 3’UTR, luciferase activity decreased following ectopic expression of miR-449a, but this was not observed in the mutant constructs (Figure 4B).

To verify these results in cells, qRT-PCR and western blotting were used to detect *Tcf3* expression in two HCC cell lines infected with Lv-miR-449a or Lv-NC. As shown in Figure 4C and 4D, TCF3 RNA and protein levels were both significantly reduced in the miR-449a overexpression group. These results indicate that miR-449a can regulate the expression of TCF3 at both the mRNA and protein levels.

In the above data, miR-449a was shown to be upregulated in cancer tissue compared to adjacent normal tissue. To determine how this upregulation affects TCF3 expression in HCC tissues, we measured levels of miR-449a and TCF3 mRNA in 25 HCC tissue samples paired with adjacent normal tissues. As expected, TCF3 expression was increased in the paired miR-449a-low-expression adjacent normal tissues. Additionally, miR-449a expression was negatively correlated with TCF3 expression (Figure 4E, upper panel). These results indicate that an inverse correlation exists between miR-449a and TCF3 expression in HCC tissue.

### TCF3 can offset the stem cell-like features and tumorigenicity of miR-449a-overexpressing cells

**Figure 5:**
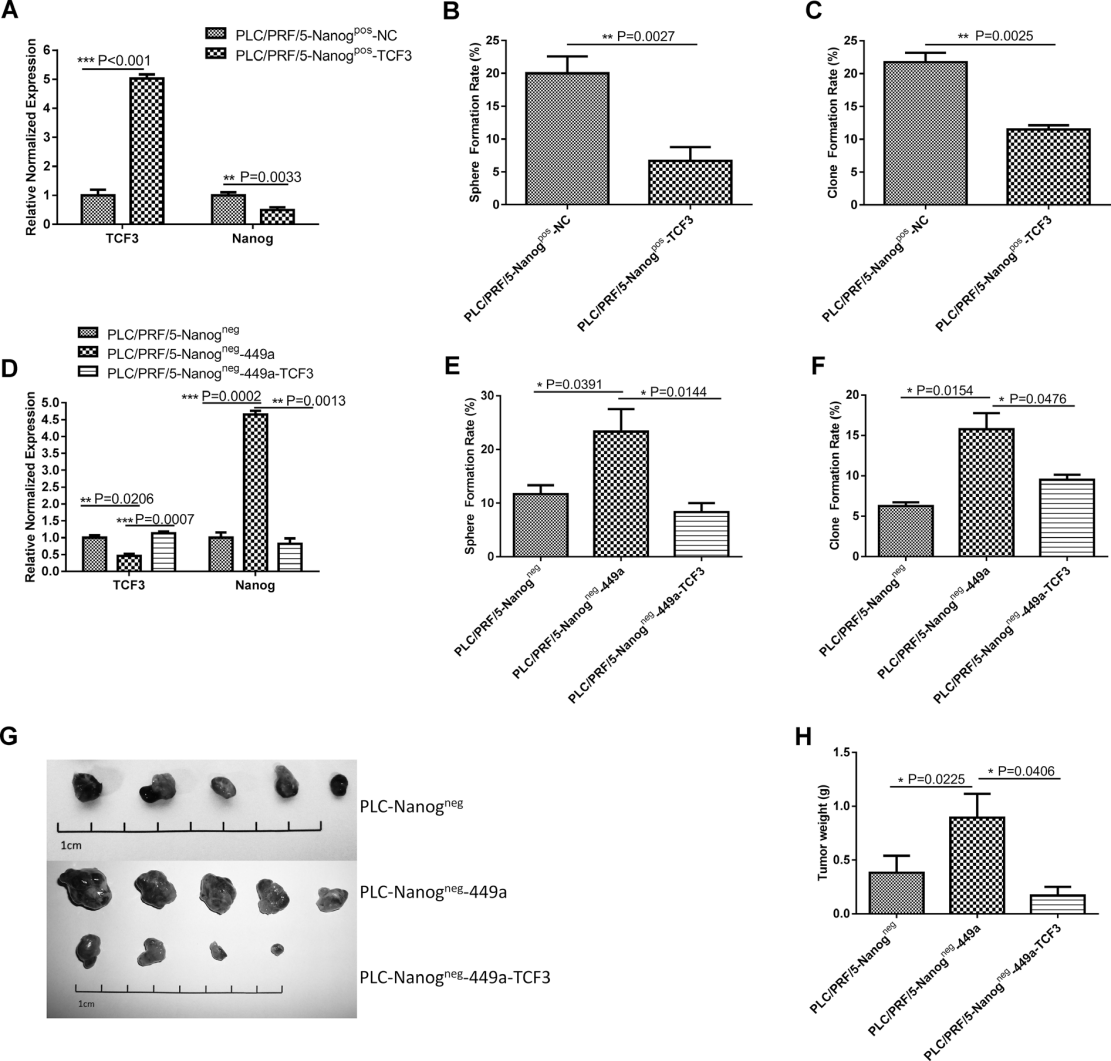
TCF3 can offset the stem cell-like features and tumorigenicity of miR-449a-overexpressing cells (**A**) TCF3 and Nanog mRNA expression in scrambled control-expressing PLC/PRF/5 Nanog^pos^ cells (Nanog^pos^-NC) and TCF3-expressing cells (Nanog^pos^-TCF3) were detected using qRT-PCR. (The data are presented as the mean ± SD of three independent experiments; ^***^P < 0.001; ^**^P < 0.01). (**B**, **C**) Sphere formation rate and clone formation rate of scrambled control-expressing PLC/PRF/5 Nanog^pos^ cells (Nanog^pos^-NC) and TCF3-expressing PLC/PRF/5 Nanog^pos^ cells (Nanog^pos^-TCF3) grown in suspension or in conventional culture conditions for 14 days. (The data are presented as the mean ± SD of three independent experiments; ^**^P < 0.01). (**D**) Expression of TCF3 and Nanog mRNA in PLC/PRF/5 Nanog^neg^ cells (Nanog^neg^), miR-449a-expressing PLC/PRF/5 Nanog^neg^ cells (Nanog^neg^-449a) and TCF3-expressing Nanog^neg^-449a cells (Nanog^neg^-449a-TCF3) detected using qRT-PCR. (The data are presented as the mean ± SD of three independent experiments; ^***^P < 0.001; ^**^P < 0.01). (**E**, **F**) Sphere formation rate and clone formation rate of Nanog^neg^, Nanog^neg^-449a and Nanog^neg^-449a-TCF3 cells grown in suspension or in conventional culture conditions for 14 days. (The data are presented as the mean ± SD of three independent experiments; ^*^P < 0.05). (**G**) *In vivo* tumor formation of Nanog^neg^, Nanog^neg^-449a and Nanog^neg^-449a-TCF3 cells. A total of 1 × 10^5^ cells were injected into NOD-SCID mice (5 mice per group). Representative xenografts from each of the five mice are shown. Lentivirus-mediated upregulation of TCF3 significantly decreased tumor formation by PLC/PRF/5 Nanog^neg^-449a cells. (**H**) Average weight of tumors formed from Nanog^neg^, Nanog^neg^-449a or Nanog^neg^-449a-TCF3 cells initiated by implantation of 1 × 10^5^ cells. Downregulation of miR-449a by TCF3 overexpression significantly reduced tumor weight. (5 mice per group, and the data are presented as the mean ± SD; ^*^P < 0.05).

It has been reported that TCF3 can bind to a promoter regulatory region of the *Nanog* gene and repress its transcriptional activity in embryonic stem cells (ESCs) through a Groucho interaction, which is a domain-dependent process. As a result, TCF3 can limit ESC self-renewal ability [24]. Therefore, to investigate whether TCF3 represses *Nanog* transcription in CSCs, we examined the mRNA expression of the *Nanog* gene in Nanog^pos^ PLC/PRF/5 cells after TCF3 overexpression. As shown in Figure 5A, TCF3 overexpression downregulated the expression of Nanog mRNA and reduced tumor sphere formation and clone formation (Figure 5B, 5C).

To determine the function of TCF3 in miR-449a-induced CSCs, we overexpressed TCF3 using lentivirus. As shown in Figure 5D, restoration of TCF3 markedly reduced the level of Nanog mRNA and the sphere formation and clone formation ability of miR-449a-overexpressing Nanog^neg^ PLC/PRF/5 cells (Figure 5E, 5F). Meanwhile, restoration of TCF3 markedly reduced the miR-449a expression (Supplementary Figure 2). Furthermore, a tumorigenicity test in NOD-SCID mice showed that restoration of TCF3 reduced the number and weight of tumors formed from miR-449a-overexpressing Nanog^neg^ PLC/PRF/5 cells (Figure 5G, 5H, Table 3).

**Table 3:** Regulation of tumorigenesis in subcutaneous xenografts by miR-449a and TCF3

Cells	Number of cells per injection
	10^5^
PLC/PRF/5 Nanog^neg^	5/5
PLC/PRF/5 Nanog^neg^-449a	5/5
PLC/PRF/5 Nanog^neg^-449a-TCF3	4/5

Note: Efficiency of tumor formation of PLC/PRF/5 Nanog^neg^ cells (PLC/PRF/5 Nanog^neg^), miR-449a-expressing PLC/PRF/5 Nanog^neg^ cells (PLC/PRF/5 Nanog^neg^-449a), and TCF3-expressing PLC-Nanog^neg^-449a cells (PLC/PRF/5 Nanog^neg^-449a-TCF3). Approximately 1 × 10^5^ cells were subcutaneously injected into NOD-SCID mice (5 mice per group). Tumor formation was observed for 4 weeks after implantation.

### miR-449a promotes the self-renewal capacity of hcc stem cells by downregulating TCF3 expression

**Figure 6:**
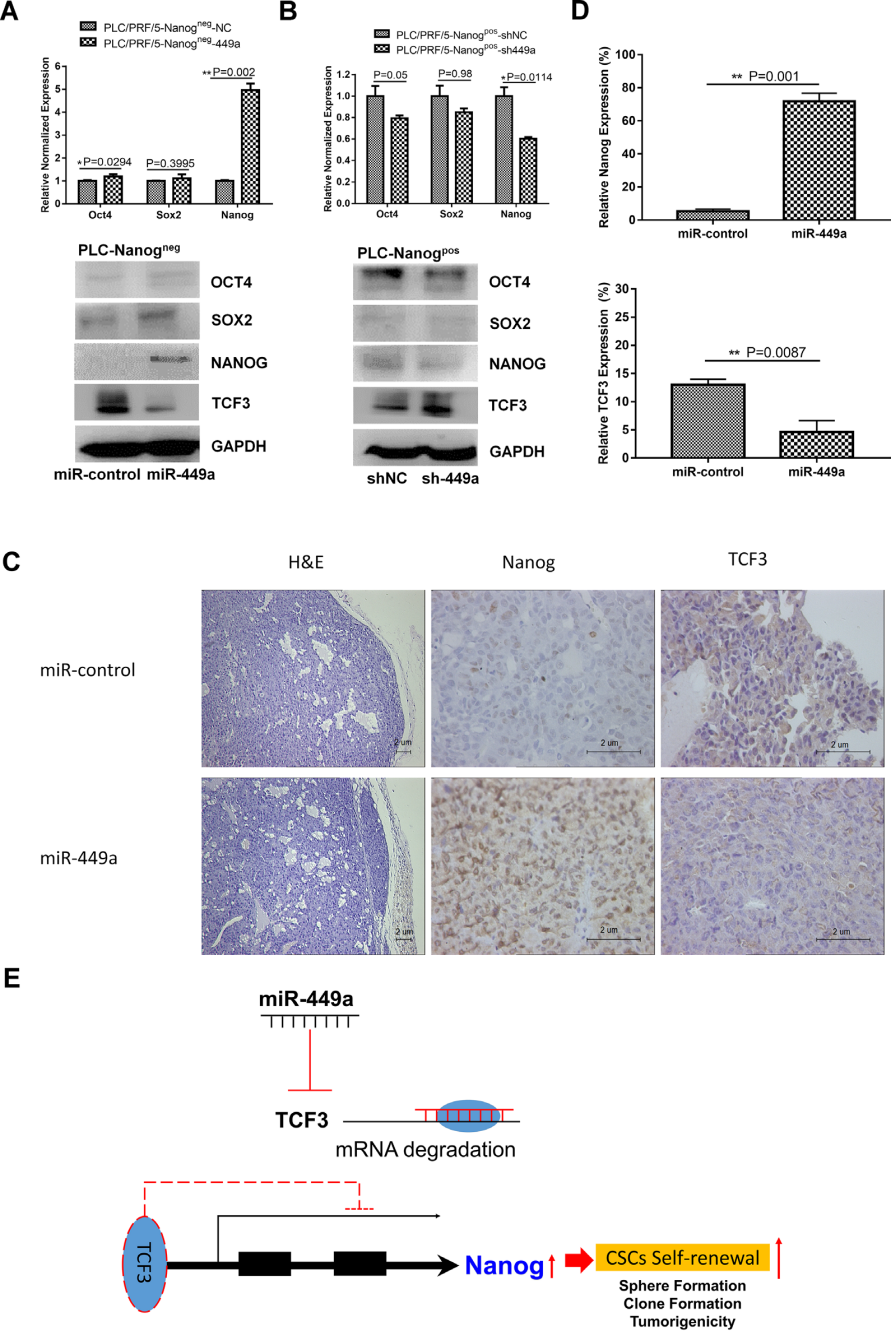
miR-449a promotes the self-renewal capacity of HCC stem cells by downregulating TCF3 expression (**A**) qRT-PCR and western blotting analysis of the expression levels of stem cell markers in PLC/PRF/5 Nanog^neg^ cells after infection with Lv-miR449a or scrambled miRNA control lentivirus. (**B**) qRT-PCR and western blotting analysis of stem cell markers in PLC/PRF/5 Nanog^pos^ cells after infection with Lv-sh-miR-449a or scrambled shRNA control lentivirus. (**C**) Histological and IHC analysis of xenografted tumors formed from miR-449a-overexpressing PLC/PRF/5 Nanog^neg^ cells or scrambled control-expressing cells. Hematoxylin and eosin staining and IHC staining with anti-Nanog or anti-TCF3 antibodies were used to examine the subcutaneous tumors (Scale bars: 2 μm). (**D**) Quantification of Nanog and TCF3 expression by cell counting in 5 randomly selected areas. (Data are presented as the mean ± SD of three independent experiments; ^**^P < 0.01). (**E**) Schema of the miR-449a-TCF3-Nanog pathway. miR-449a inhibits TCF3 expression, thereby increasing Nanog expression to maintain self- renewal in liver CSCs.

In addition to being a direct target of miR-449a, TCF3 is also a core regulator that governs the expression of genes related to stemness as well as stem cell self-renewal, differentiation, and proliferation. Therefore, we next examined the correlation between miR-449a expression and stemness markers such as Oct4, Sox2, and Nanog. To accomplish this, we overexpressed miR-449a in PLC/PRF/5 Nanog^neg^ cells and inhibited miR-449a expression in PLC/PRF/5 Nanog^pos^ cells. Then, we detected the expression of Oct4, Sox2, and Nanog using qRT-PCR and western blotting. The results showed that overexpression of miR-449a in Nanog^neg^ cells promoted the expression of the stem cell markers such as Nanog and Oct4 (Figure 6A). Conversely, inhibiting miR-449a expression in Nanog^pos^ stem cells reduced the expression of Nanog relative to that in control cells (Figure 6B). In addition, we measured the levels of miR-449a and Nanog mRNA in 25 HCC tissue samples and paired adjacent normal tissues. miR-449a expression was positively correlated with Nanog expression (Figure 4E, lower panel). These results suggest that miR-449a can increase the expression of the stemness-associated gene *Nanog*.

Immunohistochemical staining showed decreased expression of TCF3 and increased expression of Nanog in PLC/PRF/5 miR-449a-overexpressing xenografted tumors compared with control xenografted tumors, suggesting that miR-449a can also regulate HCC stemness *in vivo* (Figure 6C, 6D). Taken together, these data indicate that miR-449a improves the self-renewal capacity of HCC stem-like cells.

## DISCUSSION

Although liver CSCs and specific stem cell markers have been identified in previous studies [6, 10, 25], the molecular mechanisms by which cells acquire CSC properties such as self-renewal, drug resistance and tumor-seeding ability are not fully understood. We previously showed that Nanog plays a crucial role in maintaining the self-renewal ability of liver CSCs [10]; however, how CSCs maintain *Nanog* gene expression has not been elucidated. miRNAs have been implicated in the regulation of CSC properties; therefore, a better understanding of the modulation of gene expression in CSCs by miRNAs could aid in the identification of promising biomarkers and therapeutic targets [13, 26, 27]. In the present study, we demonstrated that miR-449a is overexpressed in HCC, especially poorly differentiated HCC, and is associated with vascular invasion and correlated with poor patient prognosis. Notably, poor differentiation, high invasion and metastasis, and poor prognosis are the characteristics of CSCs, but whether miR-449a plays a critical role in liver CSC behavior is unknown. To help address this question, we measured the expression of miR-449a in stem-like/enriched cells (tumorspheres and drug-resistant cells) and found that miR-449a was significantly upregulated in these stem-like cells. We also examined the relationship between miRNA-449a and Nanog. The transcription factor Nanog not only maintains the pluripotency and self-renewal of ESCs [28] but also helps maintain CSC stemness and promotes tumor initiation [10, 29, 30]. To better understand how miRNA-449a and Nanog are related, we detected the expression of miRNA-449a in Nanog^pos^ and Nanog^neg^ HCC cells. As expected, miRNA-449a was upregulated in the Nanog^pos^ cells.

To further investigate the function of miR-449a in stemness regulation, we artificially regulated miR-449a expression using miR-449a-expressing or anti-miR449a-expressing lentivirus in a previously described CSC Nanog reporter system [10]. In this system, Nanog^pos^ and Nanog^neg^ cells can be separated based on GFP expression, as they are all infected with a lentiviral vector containing the human *Nanog* promoter driving the expression of the GFP reporter gene. Nanog-positive HCC cells exhibited CSC-like characteristics, whereas Nanog-negative HCC cells exhibited characteristics of differentiated cells. Furthermore, after infection with the miR-449a-expression virus, Nanog^neg^ cells showed upregulated Nanog expression and exhibited restored sphere formation, enhanced clone formation and increased tumorigenicity. In contrast, after infection with the anti-miR-449a-expression virus, Nanog^pos^ cells lost their stem cell characteristics.

miR-449 and miR-34 belong to an evolutionarily conserved miRNA family [31, 32]. Previous data have demonstrated that miR-34 can induce apoptosis, cell cycle arrest and senescence in some types of cancer cells [33, 34] and can suppress the formation of gastric cancer tumorspheres [35]. However, most of the data have been derived from studies using cell culture models, and further studies should thus be conducted to investigate the potential role of miR-34 in tumors *in vivo*.

Although miR-449 miRNAs have been shown to cause cell cycle arrest or promote apoptosis in liver cancer [36, 37], the function of miR-449 in the regulation of CSCs is still unknown. Here, we showed that Nanog is not a direct target of miR-449a, which led us to search for a target gene that could regulate *Nanog* expression. We analyzed the 3’UTRs of significantly differentially expressed genes in a complementary DNA microarray and identified TCF3 as an ideal candidate. TCF3, a member of the Tcf/Lef family, is a negative regulator of the ESC pluripotency network and has also been found to inhibit pluripotency and repress *Nanog* gene expression in ESCs [24, 38]. In cancer, TCF3 inhibits embryonal carcinoma malignancy by regulating Oct4 expression [39]. In glioblastoma, downregulation of TCF3 by ASCL1 is essential for the maintenance and *in vivo* tumorigenicity of glioblastoma CSCs [40]. To demonstrate that TCF3 is a key intermediate in the upregulation of *Nanog* through miR-449a, we restored TCF3 expression in miR-449a-expressing Nanog^pos^ cells. The restoration of TCF3 abrogated the cells’ stemness features, including sphere formation, clone formation, and tumorigenicity. In addition, we demonstrated that Nanog^pos^ liver cancer cells have low expression of TCF3. Moreover, when we overexpressed TCF3 in Nanog^pos^ cells, the stemness features of these cells were reduced. Furthermore, miR-449a expression was negatively correlated with TCF3 expression and positively correlated with Nanog expression in liver cancer tissues. In contrast, the overexpression of miR-449a in Nanog^neg^ cancer cells promoted stemness-associated features (self-renewal ability and tumorigenicity), and restoring TCF3 expression in these cells negated the enhanced stemness. Finally, we verified that three predicted miR-449a target sequences exist in the 3’UTR of TCF3 using a luciferase assay.

Taken together, these results demonstrate that miR-449a is upregulated in HCC patients and leads to poor prognosis and could therefore be used as a prognostic marker for HCC. miR-449a promoted self-renewal and tumorigenesis in human HCC cells by targeting the stemness suppressor gene *Tcf3*. Furthermore, our data established the role of the miR449a-TCF3-Nanog axis in the maintenance of stemness in liver CSCs.

## MATERIALS AND METHODS

### Ethics statement

The study was approved by the Institutional Review Board of Southwest Hospital, Third Military Medical University (Chongqing, China). All patients provided written informed consent.

### Tissue samples

Fresh human hepatocarcinoma samples and paired adjacent tissues were obtained after receiving written informed consent from all patients. All patients underwent surgical resection of primary HCC at the Institute of Hepatobiliary Surgery, Southwest Hospital, Third Military Medical University.

### Cells and cell culture

The human hepatoma PLC/PRF/5 cell line was purchased from the American Type Culture Collection (Manassas, VA, http://www.atcc.org), and the Huh7 cell line was purchased from the Shanghai Cell Collection (Shanghai, China). All cells were cultured in Dulbecco's modified Eagle's medium (Gibco Invitrogen, Carlsbad, CA) supplemented with 10% fetal bovine serum (HyClone, Logan, UT), 100 U/mL penicillin, and 100 U/mL streptomycin and were maintained under 5% CO_2_ in a humidified incubator at 37°C.

### Cell sorting and flow cytometry

Flow cytometry was used to sort HCC cell lines after infection with Lv-P_Nanog_-GFP at an MOI of 10 as described previously [10]. The samples were analyzed and sorted on a BD FACS Aria II cell sorter (BD Biosciences, CA). Cell viability was assessed using 7-amino-actinomycin D (7AAD) staining to exclude dead cells. The top high (< 5%) expressing and top low (< 5%) expressing cells were sorted as Nanog^pos^ and Nanog^neg^ cells. The results were analyzed using FlowJo software (Tree Star, San Carlos, CA). The purity of the sorted cells was over 99%.

### Vector construction and reporter assays

The 3’ untranslated regions (UTRs) of different TCF3 constructs containing miR-449a-binding sites were cloned downstream of the luciferase reporter in the pMIR-REPORT vector system (Thermo Fisher Scientific, Waltham, MA). A QuikChange site-directed mutagenesis kit (Agilent Technologies, Santa Clara, CA) was used to create two point mutations in the seed region. The 3’UTRs of the TCF3 constructs and the mutated primer sequences are listed in Additional file 1, Supplementary Table 1.

PLC/PRF/5 human hepatoma cells overexpressing miR-449a or a scrambled miRNA control were cultured in 6-well plates and cotransfected with 1 μg of firefly luciferase reporter (pMIR-report) and 500 ng of Renilla luciferase reporter using Effectene transfection reagent (Qiagen, Hilden, Germany). Twenty-four hours post-transfection, firefly luciferase activity was measured using a Dual-Luciferase Assay (Promega Corporation, Madison, WI), and the results were normalized to Renilla luciferase activity according to the manufacturer's protocol.

### RNA extraction and quantitative real-time PCR

Total RNA was extracted from fresh tissues and cells using TRIzol (TaKaRa Bio Inc., Shiga, Japan) according to the manufacturer's instructions. miRNA quantification from extracted RNA was performed using TaqMan MicroRNA Assays (Applied Biosystems Inc., Waltham, MA). RT primers and TaqMan probes for miR-449a (Applied Biosystems Inc., Waltham, MA) were used for PCR, which was performed on an ABI 7900HT Fast Real-Time PCR System (Applied Biosystems Inc., Waltham, MA). miRNA levels were normalized to U6 levels. Three independent experiments were performed for each qRT-PCR analysis using three independent samples. Relative expression of miR-449 family members in tissues and hepatoma cell lines was compared to the mean expression of 10 normal liver samples using the equation RQ = 2–DDCT.

### qRT-PCR analysis of the miR-449a target gene

Total RNA was extracted from cells, and cDNA synthesis was performed with a PrimeScript RT reagent kit (TaKaRa Bio Inc., Shiga, Japan). The resulting cDNA was amplified using TCF3 primers with SYBR Premix Ex Taq II (TaKaRa Bio Inc., Shiga, Japan) with the parameters 95°C for 30 s, followed by 40 cycles of 95°C for 5 s and 60°C for 30 s. The primers for TCF3 were (forward) TCAAGGACACGAGGTCACCATC and (reverse) GGAGAAGTGGTCATTGCTGTAGG, and the primers for endogenous GAPDH were (forward) CACCCACTCCTCCACCTTTG and (reverse) CCACCACCCTGTTGCTGTAG. Melting curve analysis was performed at the end of the cycles to ensure product specificity. The relative quantity of TCF3, normalized to GAPDH, was calculated based on the equation RQ = 2–DDCT.

### Oligonucleotide synthesis and transfection

miR-449a inhibitor and scrambled control oligonucleotides were synthesized and purified by RiboBio Co. (Guangzhou, China) and had the following sequences: miR-449a inhibitor, 5′-ACCAGCUAACAAUACACUGCCA-3’ and scrambled control, 5′-UUCUCCGAACGUGUCACGUTT-3’. The oligonucleotides were transfected into cultured cells using Effectene transfection reagent (Qiagen, Hilden, Germany) following the manufacturer's instructions. Transfection efficiency was determined using TaqMan-based qRT-PCR (Applied Biosystems Inc., Waltham, MA).

### Immunohistochemical staining

Formalin-fixed, paraffin-embedded tumor tissues and xenografts were cut into 5-μm-thick sections and subjected to standard immunohistochemistry (IHC) using a Dako REAL EnVision Detection System (Dako Denmark A/S, Glostrup, Denmark) according to the manufacturer's guidelines. The following primary antibodies were used in this study: a rabbit polyclonal anti-Nanog antibody (Abcam, Cambridge, MA) and a mouse monoclonal anti-TCF3 antibody (R&D systems, Minneapolis, MN). Qualitative analysis of Nanog and TCF3 expression was performed independently by two pathologists as described previously.

### Sphere formation assay

To assay sphere formation efficiency, single cells were sorted and plated at 10 cells/well into ultra-low-attachment 96-well plates (Corning Inc., NY). The cells were cultured in DMEM/F12 medium (Sigma-Aldrich, St. Louis, MO) with B27 supplement (Gibco, Grand Island, NY), antibiotics, 20 ng/mL epidermal growth factor (PeproTech Inc., Rocky Hill, NJ), 20 ng/mL basic fibroblast growth factor (PeproTech Inc., Rocky Hill, NJ), and 10 ng/mL hepatocyte growth factor (PeproTech Inc., Rocky Hill, NJ). Next, 1% methylcellulose (Sigma-Aldrich, St. Louis, MO) was added to prevent cell aggregation, and individual spheres derived from a single cell were confirmed. After 4–5 days, equal volumes of fresh medium were added. The cells were incubated for 2 weeks, and spheres with a diameter > 75 μm were counted.

### Colony formation assays

Briefly, 100 cells were seeded into 24-well plates and cultured for 14 days. Colonies were fixed with 4% formaldehyde and stained with 0.1% crystal violet (Sigma-Aldrich, St. Louis, MO), and the colonies were counted.

### Tumor xenografts

Four-week-old male non-obese diabetic severe combined immunodeficient (NOD-SCID) mice were maintained in pathogen-free conditions at the animal facility of the Third Military Medical University and received humane care according to the criteria outlined in the “Guide for the Care and Use of Laboratory Animals” prepared by the National Academy of Sciences. Different numbers of cells overexpressing the scrambled miRNA control or miR-449a were resuspended in serum-free medium and mixed with Matrigel at a ratio of 1:1. The cells were then injected subcutaneously into NOD-SCID mice. The subcutaneous tumor-bearing mice were killed 8 weeks after implantation. The weight of the subcutaneous tumors was calculated, and the tumors were collected, fixed in formalin, paraffin-embedded, sectioned and stained with hematoxylin and eosin.

### Statistical analysis

All data are presented as the mean ± standard deviation. Student's *t*-test was used for comparisons of two groups. Analysis of variance was used for analysis of clinical variables. Kaplan-Meier's method was used for survival analysis. *P* < 0.05 was considered significant and is marked with an asterisk. *P* < 0.01 was considered highly significant and is marked with a double asterisk. *P* < 0.001 was considered highly significant and is marked with three asterisks.

## SUPPLEMENTARY MATERIALS FIGURES AND TABLE



## Abbreviations

CSCs: cancer stem cells
HCC: hepatocellular carcinoma
ESCs: embryonic stem cells
miRNAs: microRNAs
miR-449: microRNA-449
Nanog: Nanog homeobox
TCF3: transcription factor 3
DMEM: Dulbecco's modified Eagle's medium
FBS: fetal bovine serum
FACS: fluorescence-associated cell sorting
7AAD: 7-amino-actinomycin D
GFP: green fluorescent protein
qRT-PCR: quantitative real-time polymerase chain reaction
IHC: immunohistochemistry
OCT4: octamer-binding transcription factor 4
Sox2: sex determining region Y (SRY)-box 2
UTR: untranslated region
shRNA: short hairpin RNA
