# Supplementary material for: MicroRNA-449a maintains self-renewal in liver cancer stem-like cells by targeting Tcf3
Source: Oncotarget. 2017 Nov 27;8(66):110187–200. doi: 10.18632/oncotarget.22705 (PMC5746375; doi:10.18632/oncotarget.22705)
Supplement: Supplementary file 1 [file oncotarget-08-110187-s001.pdf]

MicroRNA-449a maintains self-renewal in liver cancer stem-like cells by targeting *Tcf3*

SUPPLEMENTARY MATERIALS

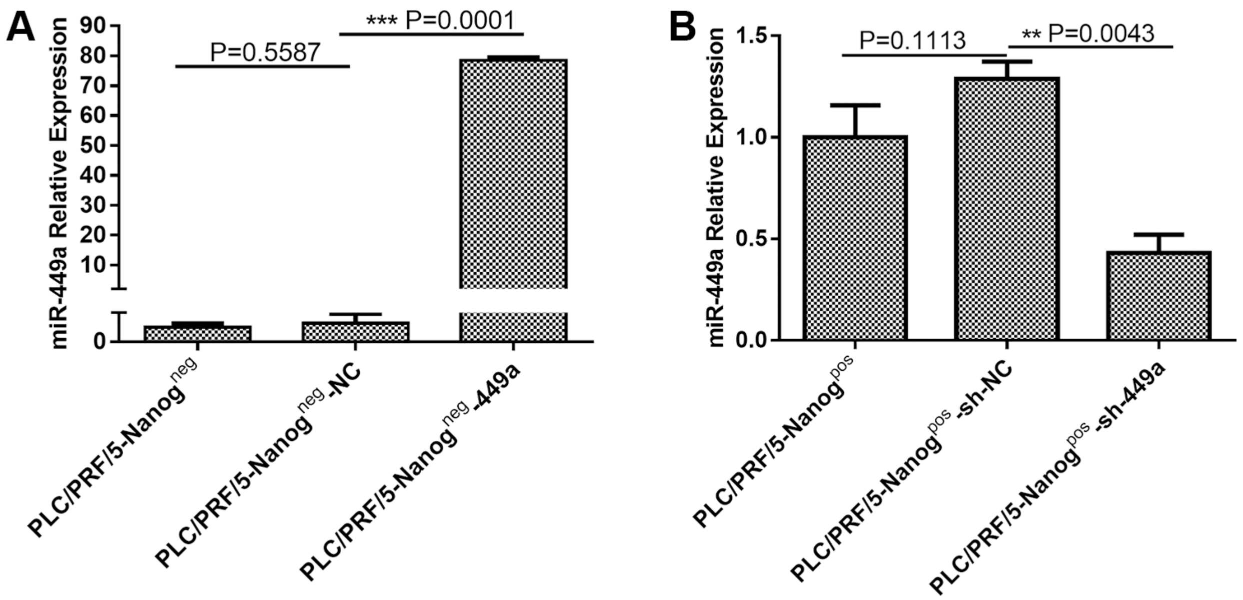

Supplementary Figure 1: Expression of miR-449a in different groups of cells. (**A**) qRT-PCR was used to detect the expression of miR-449a in PLC/PRF/5 Nanogneg cells after infection with Lv-miR449a or scrambled control miRNA lentiviruses. (Data are presented as the mean ± SD of three independent experiments; $^{***}P < 0.001$). (**B**) qRT-PCR was used to detect the expression of miR-449a in PLC/PRF/5 Nanogpos cells after infection with Lv-sh-miR-449a or scrambled control shRNA lentiviruses. (Data are presented as the mean ± SD of three independent experiments; $^{**}P < 0.01$).

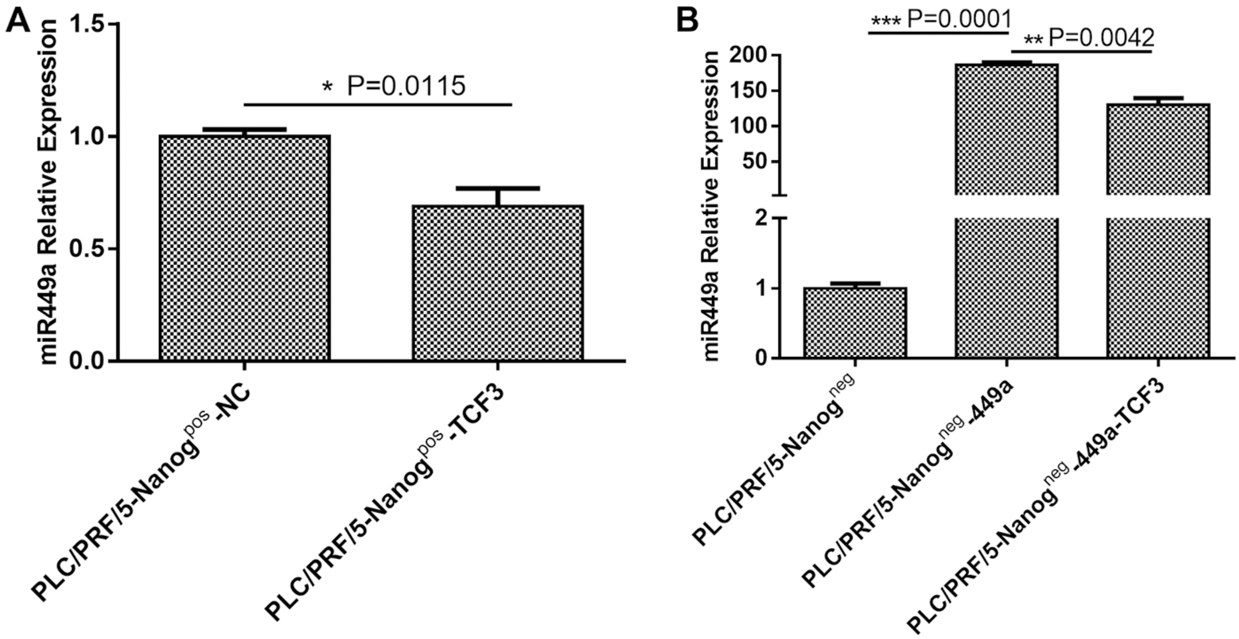

Supplementary Figure 2: Expression of miR-449a in different groups of cells. (**A**) qRT-PCR was used to detect the expression of miR-449a in scrambled control-expressing PLC/PRF/5 Nanogpos cells (Nanogpos-NC) and TCF3-expressing cells (Nanogpos-TCF3). (Data are presented as the mean ± SD of three independent experiments; $^{*}P < 0.05$). (**B**) qRT-PCR was used to detect the expression of miR-449a in PLC/PRF/5 Nanogneg cells (Nanogneg), miR-449a-expressing PLC/PRF/5 Nanogneg cells (Nanogneg-449a) and TCF3-expressing Nanogneg-449a cells (Nanogneg-449a-TCF3). (Data are presented as the mean ± SD of three independent experiments; $^{***}P < 0.001$; $^{**}P < 0.01$).

Supplementary Table 1: The 3'UTRs of TCF3 constructs and mutated primer sequences

Construct	Sequence
TCF3-3'UTR-1 positive strand	cgcgtCAGCTTCCCCGACTCCATCTGCAGCTCTGCCATTGTGACATTTCCTGTTACa
TCF3-3'UTR-1 negative strand	CTAGTgtaacaggaaatgtcacaatggcagagctgcagatggagtcggggaagctgA
TCF3-3'UTR-2 positive strand	cgcgtTTTAAAGGGACTCAAGGTGCCTGCCACTTCCTCAGCGAAGAAGTCTGTa
TCF3-3'UTR-2 negative strand	CTAGTacagacttcttcgctgaggaagtggcaggcaccttgagtccctttaaaA
TCF3-3'UTR-3 positive strand	cgcgtCTGGGTCCGTGCCAGCACAATCTGCCAAAGTTCTAGAGACCCTGTTCCCTTa
TCF3-3'UTR-3 negative strand	CTAGTaagggaacagggtctctagaactttggcagattgtgctggcacggacccagA
TCF3-3'UTR-1 mutated positive strand	cgcgtCAGCTTCCCCGACTCCATCTGCAGCTCTattATTGTGACATTTCCTGTTACa
TCF3-3'UTR-1 mutated negative strand	CTAGTgtaacaggaaatgtcacaatAATagagctgcagatggagtcggggaagctg
TCF3-3'UTR-2 mutated positive strand	cgcgtTTTAAAGGGACTCAAGGTGCCTattACTTCCTCAGCGAAGAAGTCTGTa
TCF3-3'UTR-2 mutated negative strand	CTAGTacagacttcttcgctgaggaagtAATaggcaccttgagtccctttaaaA
TCF3-3'UTR-3 mutated positive strand	cgcgtCTGGGTCCGTGCCAGCACAATCTattAAAGTTCTAGAGACCCTGTTCCCTTa
TCF3-3'UTR-3 mutated negative strand	CTAGTaagggaacagggtctctagaactttAATagattgtgctggcacggacccagA
